# Building a near-infrared (NIR) soil spectral dataset and predictive machine learning models using a handheld NIR spectrophotometer

**DOI:** 10.1016/j.dib.2024.111229

**Published:** 2024-12-16

**Authors:** Colleen Partida, Jose Lucas Safanelli, Sadia Mannan Mitu, Mohammad Omar Faruk Murad, Yufeng Ge, Richard Ferguson, Keith Shepherd, Jonathan Sanderman

**Affiliations:** a Woodwell Climate Research Center, 149 Woods Hole Rd., Falmouth, MA, 02540, United States; b Department of Biological Systems Engineering, University of Nebraska-Lincoln, E Campus Mall, Lincoln, NE, 68583, United States; c USDA, Natural Resources Conservation Service (NRCS), National Soil Survey Center (NSSC), Kellogg Soil Survey Laboratory (KSSL), 1121 Lincoln Mall, Lincoln, NE, 68508, United States; d Innovative Solutions for Decision Agriculture (iSDA), Rothamsted Campus, West Common, Harpendedn AL5 2JQ, UK

**Keywords:** Soil spectroscopy, Soil organic carbon, Pedometrics, Chemometrics, Soil analysis

## Abstract

This near-infrared spectral dataset consists of 2,106 diverse mineral soil samples scanned, on average, on six different units of the same low-cost commercially available handheld spectrophotometer. Most soil samples were selected from the USDA NRCS National Soil Survey Center-Kellogg Soil Survey Laboratory (NSSC-KSSL) soil archives to represent the diversity of mineral soils (0–30 cm) found in the United States, while 90 samples were selected from Ghana, Kenya, and Nigeria to represent available African soils in the same archive. All scanning was performed on dried and sieved (<2 mm) soil samples. Machine learning predictive models were developed for soil organic carbon (SOC), pH, bulk density (BD), carbonate (CaCO3), exchangeable potassium (Ex. K), sand, silt, and clay content from their spectra in the R programming language using most of this dataset (1,976 US soils) and are included in this data release. Two model types, Cubist and partial least squares regression (PLSR) were developed using two strategies: (1) using an average of the spectral scans across devices for each sample and, (2) using the replicate spectral scans across devices for each sample. We present the internal performance of these models here. The dry spectra and Cubist models for these soil properties are available for download from 10.5281/zenodo.7586621. An example of detailed code used to produce these models is hosted at the Open Soil Spectral Library, a free service of the Soil Spectroscopy for the Global Good Network (soilspectroscopy.org), enabling broad use of these data for multiple soil monitoring applications.

Specifications Table

**Table:** 

Subject	Soil Science
Specific subject area	Soil spectroscopy, NIR spectroscopy, predictive machine-learning modeling
Type of data	Raw Code files
Data collection	A set of 2,106 mineral soil samples were selected and scanned for inclusion in this dataset on NeoSpectra handheld NIR spectrophotometers (Si-Ware Systems, Cairo, Egypt). 2,016 samples were selected to represent mineral topsoil (0-30cm) in the US, and 90 samples from Ghana, Kenya, and Nigeria were selected to represent African soils. A subset of 1,976 US samples was used to build predictive machine learning models. Two model types (Cubist and partial least squares regression) were employed to produce predictions for eight soil properties.
Data source location	Countries: United States of America, Ghana, Kenya, and Nigeria.
Data accessibility	Repository name: Zenodo Data identification number: 10.5281/zenodo.7586621 Direct URL to data: https://zenodo.org/doi/10.5281/zenodo.7586621 Detailed code associated with the model building is available from the Github repository https://github.com/soilspectroscopy/ossl-models
Related research article	Mitu, S. M., Smith, C., Sanderman, J., Ferguson, R. R., Shepherd, K., & Ge, Y., Evaluating consistency across multiple NeoSpectra (compact Fourier transform near‐infrared) spectrometers for estimating common soil properties. Soil Science Society of America Journal Volume 88 Issue 4 (2024) 1324-1339. https://doi.org/10.1002/saj2.20678

## 1 Value of the Data

• Large soil spectral libraries typically produce reliable predictions across a range of soil types but take a large investment of time and effort to produce, thus, representing a significant barrier to entry to the use of soil spectroscopy. This spectral library and predictive models can immediately be used by anyone with a compatible NIR spectrophotometer.
• This methodology and code can be reproduced by other researchers to make predictions for bulk density (BD), calcium carbonate (CaCO3), clay content, exchangeable potassium (Ex. K), pH, sand content, silt content, and soil organic carbon (SOC) on external sample sets.
• The data extends the concept of the Global Soil Spectral Library and Estimation Service (Shepherd et al., 2023) to include NIR by selecting a diverse set of samples from the same soil spectral library and supplementing it with African soils.
• We hope that by providing this spectral library, other researchers can apply more advanced modeling techniques that can add value, and even potentially replace, the models that are provided in this data release.

## 2 Background

Diffuse reflectance infrared spectroscopy has become an indispensable laboratory tool for rapid estimation of numerous soil properties to support soil mapping, soil monitoring and soil testing applications [4]. Recent advances in hardware technology have enabled the development of handheld sensors with similar performance specifications as laboratory-grade near infrared (NIR) spectrophotometers [6]. Handheld sensors like the NeoSpectra scanner used in this dataset are more cost effective than traditional laboratory grade spectrometers. By building and publicly providing a library of spectral data with associated quality-controlled analytical data on numerous soil properties and predictive models, we are offering researchers and soil professionals the ability to utilize this dataset and predictive models to make predictions for soil properties on scans of their own samples.

## 3 Data Description

The spectral dataset is provided in two formats:

1) The file “Neospectra_WoodwellKSSL_soil+site+NIR.csv” includes six individual spectral scans from each of approximately five different scanners per sample.
2) The subset of 1,976 samples used to build the models described below is provided in the file “1976_NSlibrary_withmetadata.csv”. In this file, each row contains the averaged spectra for a given scanner and soil sample (1 spectra per scanner per soil sample).

The Cubist models as presented and described here are provided as Quick serialization (“.qs”) files (Table 1). This data format is accessible for use in the R programming language.

**Table 1:** Model file names and descriptions for the Cubist average (one average spectra per sample) each sample, so there is only one spectra per sample) and Cubist replicate (one spectra per scanner per sample) models for 8 soil properties.

File description	File name
Cubist average model for log(1+BD).	log..bd_model_nir.neospectra_cubist_AVG_ossl_na_v1.2.qs
Cubist average model for log(1+CaCO3).	log..caco3_model_nir.neospectra_cubist_AVG_ossl_na_v1.2.qs
Cubist average model for clay.	clay_model_nir.neospectra_cubist_AVG_ossl_na_v1.2.qs
Cubist average model for log(1+Ex. K).	log..k.ex_model_nir.neospectra_cubist_AVG_ossl_na_v1.2.qs
Cubist average model for pH.	ph.h2o_model_nir.neospectra_cubist_AVG_ossl_na_v1.2.qs
Cubist average model for sand.	sand_model_nir.neospectra_cubist_AVG_ossl_na_v1.2.qs
Cubist average model for silt.	silt_model_nir.neospectra_cubist_AVG_ossl_na_v1.2.qs
Cubist average model for log(1+SOC).	log..soc_model_nir.neospectra_cubist_AVG_ossl_na_v1.2.qs
Cubist replicate model for log(1+BD).	log..bd_model_nir.neospectra_cubist_REPS_ossl_na_v1.2.qs
Cubist replicate model for log(1+CaCO3).	log..caco3_model_nir.neospectra_cubist_REPS_ossl_na_v1.2.qs
Cubist replicate model for clay.	clay_model_nir.neospectra_cubist_REPS_ossl_na_v1.2.qs
Cubist replicate model for log(1+Ex. K).	log..k.ex_model_nir.neospectra_cubist_REPS_ossl_na_v1.2.qs
Cubist replicate model for pH.	ph.h2o_model_nir.neospectra_cubist_REPS_ossl_na_v1.2.qs
Cubist replicate model for sand.	sand_model_nir.neospectra_cubist_REPS_ossl_na_v1.2.qs
Cubist replicate model for silt.	silt_model_nir.neospectra_cubist_REPS_ossl_na_v1.2.qs
Cubist replicate model for log(1+SOC).	log..soc_model_nir.neospectra_cubist_REPS_ossl_na_v1.2.qs

An example of detailed code to produce and run the Cubist average models is available from the Github repository (https://github.com/soilspectroscopy/ossl-models/), allowing for predictions to be reproduced. The corresponding code files for this analysis are all annotated with the model name “nir.neospectra_cubist_ossl_na_v1.2”.

## 4 Experimental Design, Materials and Methods

### 4.1 Sample selection

From a previous project [7], 519 US samples were queried from the USDA NRCS NSSC-KSSL soil archives as having a complete set of eight measured properties (total carbon, total organic carbon, total nitrogen, cation exchange capacity, pH, clay, sand, and silt). They were stratified based on the major horizon and taxonomic order, omitting the categories with less than 500 samples. Three percent of each stratum (i.e., a combination of major horizon and taxonomic order) was then randomly selected as the final subset retrieved from KSSL's physical soil archive as 2-mm sieved samples. With a goal of building a dataset of 2000 samples, additional US samples were queried from the USDA NRCS NSSC-KSSL soil archives with the following criteria described in Mitu et al. [1]: lower depth ≤ 30 cm, pH range 4.0 to 9.5, organic carbon <10 %, greater than lower detection limits for all properties, actual physical samples available in the archive, samples collected and analyzed from 2001 onwards, samples having complete analyses for high-priority properties (sand, silt, clay, cation exchange capacity, Buffered ammonium-acetate exchangeable Ca, Mg, K and Na, and SOC), and MIR scanned. Out of all samples meeting these criteria (>20,000), Latin hypercube sampling was used to limit this new set to 1,497 samples. Additionally, 90 samples from Ghana, Kenya, and Nigeria were selected from the archives and scanned to represent African soils available in the archive. Summary statistics for the 2,106 samples in this dataset are listed in Table 2 below.

**Table 2:** Summary statistics of 8 soil properties for the samples in this dataset.

Soil property	count	Mean	Minimum	Q1*	Median	Q3⁎⁎	Maximum	Lab method (KSSL method code)
SOC (%)	2106	2	-0.03	0.59	1.31	2.63	53.88	Total carbon (4H2a1) minus inorganic carbon (4E1a1a1)
pH	2096	6.25	3.69	5.21	6.12	7.35	9.52	1:1 water extraction (4C1a2a1)
BD (g/cm3)	964	1.31	0.3	1.18	1.34	1.47	2.03	Clod (1B1a2)
CaCO3 (%)	693	5.91	-0.57	0.26	1.64	7.61	89.03	HCl treatment/manometric (4E1a1a1)
Ext. K (cmol(+) kg^−1^)	2096	0.55	0	0.14	0.33	0.68	11.25	NH4OAc/pH 7 extraction (4B1a1c1-4)
Sand (%)	2106	41.88	0.3	17.6	39.25	64.38	100	Pipette method (3A1a)
Silt (%)	2106	37.57	0	21.8	37.4	52.1	87.9	Pipette method (3A1a)
Clay (%)	2106	20.55	0	9.08	18.33	28.83	86.69	Pipette method (3A1a)

⁎ Q1 = Quartile 1 (Lower Quartile)

⁎⁎ Q3 = Quartile 3 (Upper Quartile)

### 4.2 Scanning and lab methods

The selected dry 2-mm sieved soil samples were then scanned using NeoSpectra handheld NIR spectrophotometers (Si-Ware Systems, Cairo, Egypt). These NeoSpectra scanners use an internal light source to capture spectral reflectance within the NIR range of 1350–2500 nm, collecting 257 data points with linear interpolation and 32k fast Fourier transform points for each measurement [1].

**Fig. 1:**
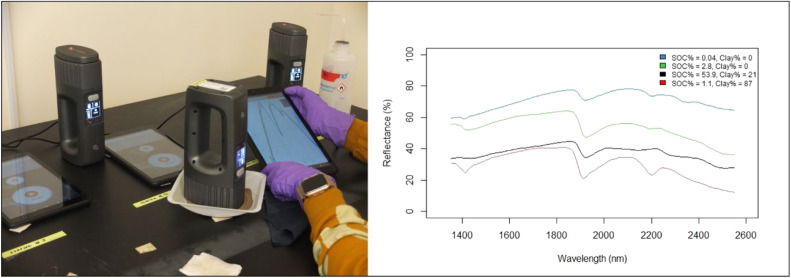
A photo of the scanning process with multiple scanners (left), and a plot of raw spectra from 4 samples of differing reflectance (right).

To scan a sample, approximately 20–50 g of dry soil sieved to < 2 mm was added to a plastic weigh boat. The optical surface of the scanner (10 mm in diameter) was placed in contact with the soil surface, and six individual scans were taken as the optical window was moved slowly across the surface of the sample. To capture instrument variability, nine separate NeoSpectra scanners were used throughout the course of the study as functional issues necessitated the replacement of a few (Fig. 1). The aim was to scan each soil sample on at least five different scanners, this was completed as possible and the scans with corresponding instrument serial numbers can be found in the linked Zenodo repository. Of the 2,106 total soil samples, 519 samples were scanned following this protocol at Woodwell Climate Research Center and the remaining 1,587 samples were scanned at the NSSC-KSSL utilizing the same protocol.

All analytical data on the various soil properties were generated by the KSSL following published methods [5]. The method codes are available in Table 2.

### 4.3 Model building

These six scans per sample per scanner were averaged to create one scan per sample on each individual scanner, and a large subset of the data containing 1,976 unique US soil samples was used for model building. The spectra in reflectance units were preprocessed by interpolating to 2 nm intervals, increasing the number of wavelength columns from 257 to 601 and a Standard Normal Variate (SNV) transformation was applied. Two machine learning algorithms, Cubist (using the R package ‘mlr3’) and Partial Least Squares Regression (PLSR; using the R package ‘mdatools’), were employed to develop predictive models for eight soil properties, including bulk density (BD), calcium carbonate (CaCO3), clay content, buffered ammonium-acetate exchangeable potassium (Ex. K), pH, sand content, silt content, and soil organic carbon (SOC). Except for the granulometric fractions and pH, all soil properties were natural log transformed with an offset (log(1+x)) before model calibration to control the skewness of the range of values. Performance metrics are presented using the log(1+x) units.

Two strategies were evaluated for handling scanner-to-scanner variability: averaging the scans across scanners for each sample (avg) versus retaining scans from each scanner for each sample (reps) during model building. In both strategies the six replicate scans on each scanner for each sample were averaged prior to any model building.

PLSR is a standard algorithm in chemometrics [8] and predictive models were built testing up to 30 factors with the number of factors being optimized by 5-fold cross-validation. Internal evaluation of the dataset was done separately by refitting the fine-tuned models with 10-fold cross-validation. Cubist models [2], in turn, were built using the Open Soil Spectral Library (OSSL) framework which consists of compressing the spectra up to the n first components retaining around 99.99 % of the original cumulative variance in the spectra and employing the components scores as training features [3]. Cubist models were fine-tuned, setting 0 for the hyperparameter “neighbors” and testing an optimum number of “committees” in the range of [1,5,10,15,20] using 5-fold cross-validation. Similarly, 10-fold cross-validation with refitting was used for internal model evaluation. For the replicate models, random splits of cross-validation were made by ensuring the soil sample IDs were grouped/blocked together to avoid information leakage and over-optimistic performance estimation.

### 4.4 Model evaluation

**Fig. 2:**
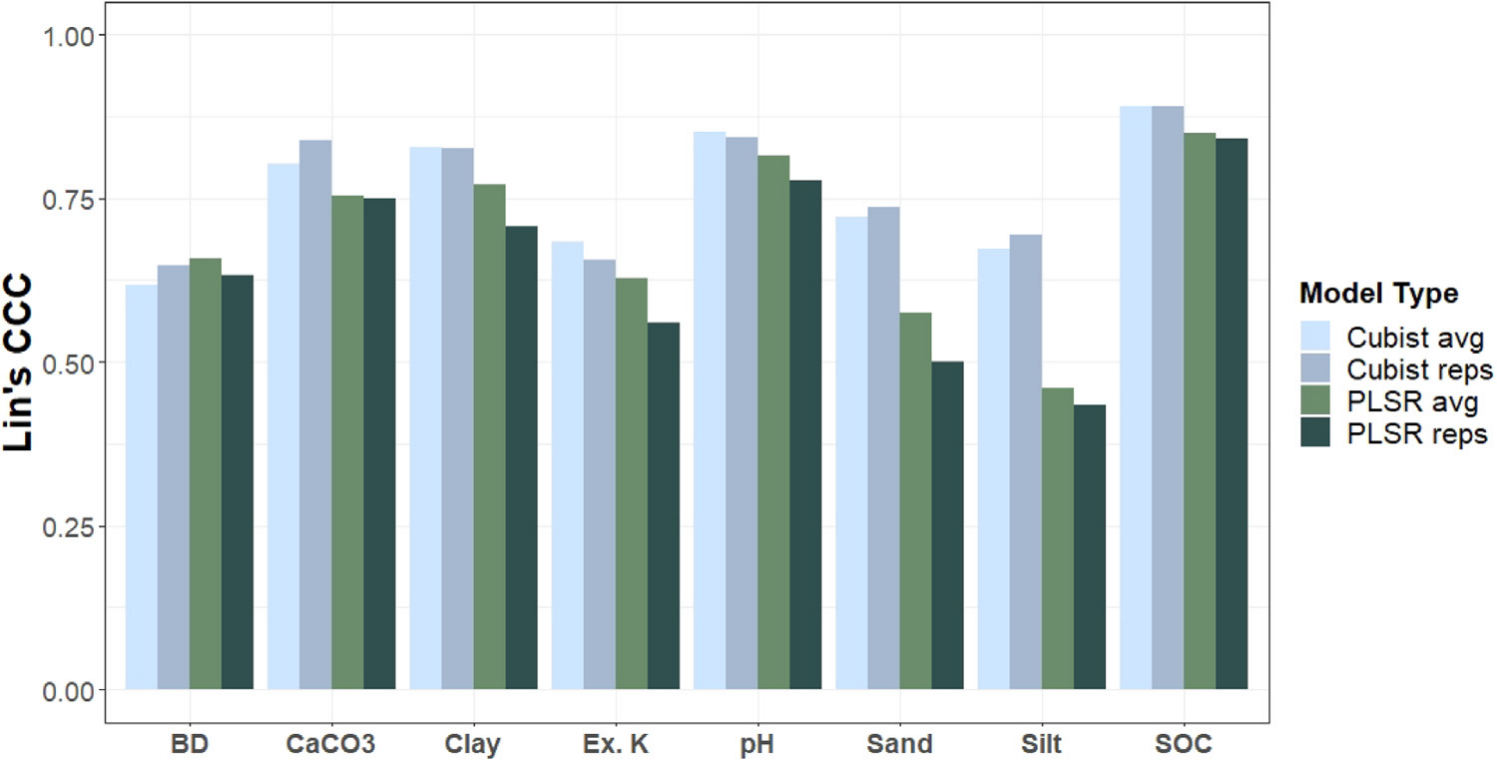
Lin's CCC values for the 4 model types across all 8 properties.

**Fig. 3:**
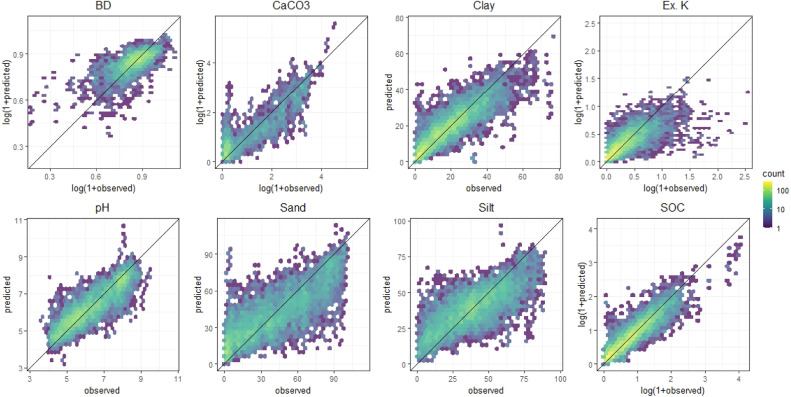
Goodness of fit plots for the Cubist replicate models from top left to right for bulk density (BD), calcium carbonate (CaCO3), clay content, exchangeable potassium (Ex. K), and from bottom left to right for pH, sand content, silt content, and soil organic carbon (SOC).

Models were evaluated internally using a 10-fold cross-validation with a refitting approach to assessing their performance in predicting soil properties. The performance metrics included Root Mean Square Error (RMSE), mean error (bias), the square of the correlation coefficient (R²), Lin's Concordance Correlation Coefficient (CCC), and the ratio of performance to interquartile distance (RPIQ). Cubist models had larger CCC values than the PLSR models for all soil properties except BD, with the models built on averaged spectra slightly outperforming models built using the replicate scans for most soil properties (Tables 3 & 4). The best models for SOC, CaCO3, clay, and pH all had CCC values > 0.80. Sand, silt, exchangeable K, and BD all had slightly lower Lin's CCC between 0.66 and 0.74 for the best models (Fig. 2). These performance metrics, also summarized in goodness-of-fit plots (Fig. 3), are presented to justify the inclusion of only the Cubist models in the code repository. Both the average and replicate models have been included because we believe the replicate models might outperform the average models when applied in a new setting with a scanner different than one of the nine used in building this database.

**Table 3:** Model performance statistics for the Cubist models.

Soil property	Variant	Unit	n	RMSE	bias	R^2^	CCC	RPIQ
BD	AVG	log(1+x)	1085	0.08	-0.01	0.45	0.62	1.46
BD	REPS	log(1+x)	4157	0.08	0.00	0.47	0.65	1.49
CaCO3	AVG	log(1+x)	665	0.64	-0.02	0.67	0.80	3.06
CaCO3	REPS	log(1+x)	2705	0.60	-0.01	0.73	0.84	3.55
Clay	AVG	original	1976	7.30	0.12	0.72	0.83	2.60
Clay	REPS	original	7790	7.47	0.13	0.72	0.83	2.56
K	AVG	log(1+x)	1976	0.22	0.03	0.54	0.68	1.75
K	REPS	log(1+x)	7790	0.23	0.02	0.49	0.66	1.63
pH	AVG	original	1976	0.64	0.01	0.75	0.85	3.39
pH	REPS	original	7790	0.66	0.01	0.73	0.84	3.37
Sand	AVG	original	1976	18.16	0.63	0.59	0.72	2.58
Sand	REPS	original	7790	18.29	0.20	0.59	0.74	2.66
Silt	AVG	original	1976	13.79	0.18	0.54	0.67	2.17
Silt	REPS	original	7790	13.98	-0.07	0.54	0.69	2.18
SOC	AVG	log(1+x)	1974	0.25	-0.01	0.80	0.89	3.22
SOC	REPS	log(1+x)	7782	0.26	0.00	0.81	0.89	3.29

**Table 4:** Model performance statistics for the PLSR models.

Soil property	Variant	Unit	n	RMSE	bias	R^2^	CCC	RPIQ
BD	AVG	log(1+x)	1085	0.08	0.00	0.47	0.66	1.50
BD	REPS	log(1+x)	4157	0.08	0.00	0.45	0.63	1.45
CaCO3	AVG	log(1+x)	665	0.74	-0.01	0.57	0.75	2.66
CaCO3	REPS	log(1+x)	2705	0.75	0.01	0.58	0.75	2.84
Clay	AVG	original	1976	8.52	-0.04	0.62	0.77	2.23
Clay	REPS	original	7790	9.57	-0.02	0.53	0.71	2.00
K	AVG	log(1+x)	1976	0.24	0.00	0.44	0.63	1.59
K	REPS	log(1+x)	7790	0.25	0.00	0.37	0.56	1.47
pH	AVG	original	1976	0.72	0.00	0.68	0.81	3.02
pH	REPS	original	7790	0.78	0.00	0.63	0.78	2.87
Sand	AVG	original	1976	22.30	0.03	0.38	0.57	2.10
Sand	REPS	original	7790	23.69	-0.03	0.31	0.50	2.05
Silt	AVG	original	1976	17.10	0.00	0.28	0.46	1.75
Silt	REPS	original	7790	17.83	0.01	0.26	0.43	1.71
SOC	AVG	log(1+x)	1974	0.29	0.00	0.73	0.85	2.76
SOC	REPS	log(1+x)	7782	0.31	0.00	0.72	0.84	2.75

## Limitations

• While this spectral library is broadly representative of the diversity of 0–30 cm mineral soils found in the USA, specific soil types might be underrepresented and as such users are encouraged to check for spectral similarity with their samples before applying these models.
• The library was built by scanning each sample on an average of five different Neospectra scanners to try to ensure that variability across scanners are captured, but these data and models have not been tested against NIR spectra obtained on different brands of scanners.
• This library will not be appropriate for soil samples scanned under field conditions because all samples were dried and sieved prior to scanning.
• Compared to wet chemistry methods, NIR spectral predictions add uncertainty to soil property estimates and users need to consider if this increased uncertainty outweighs the benefits of rapid low-cost soil monitoring.
• The Open Soil Spectral Library is a living resource with the library itself growing and models being updated periodically.

## Ethics Statement

The authors of this dataset have read and followed the ethical requirements for publication in Data in Brief, and confirm that this work did not involve human subjects, animal experiments, or any data collected from social media platforms.

## Credit Author Statement

**Colleen Partida:** Writing - Original Draft, Data Curation, Formal analysis, Investigation. **Jose Lucas Safanelli:** Methodology, Data Curation, Writing - Review & Editing. **Sadia Mannan Mitu:** Investigation, Data Curation. **Mohammad Omar Faruk Murad:** Investigation. **Yufeng Ge:** Supervision, Conceptualization. **Richard Ferguson:** Resources, Supervision, Conceptualization. **Keith Shepherd:** Funding acquisition, Supervision, Conceptualization. **Jonathan Sanderman:** Supervision, Conceptualization, Project administration, Writing - Original Draft.

## Data Availability

ZenodoNear-infrared (NIR) soil spectral library using the NeoSpectra Handheld NIR Analyzer by Si-Ware (Original data).
